# Balancing capacity and epidemic spread in the global airline network

**DOI:** 10.1007/s41109-021-00432-0

**Published:** 2021-11-25

**Authors:** Robert Harper, Philip Tee

**Affiliations:** 1 Science Group, Moogsoft Ltd., London, UK; 2 Science Group, Moogsoft Inc., San Francisco, CA USA; 3 The Beyond Center for Fundamental Science, University of Arizona, Tempe, AZ USA; 4 Department of Informatics, University of Sussex, Falmer, Brighton, UK

**Keywords:** COVID-19, Epidemic spread, Vertex entropy, Complex networks, Transport networks

## Abstract

The structure of complex networks has long been understood to play a role in transmission and spreading phenomena on a graph. Such networks form an important part of the structure of society, including transportation networks. As society fights to control the COVID-19 pandemic, an important question is how to choose the optimum balance between the full opening of transport networks and the control of epidemic spread. In this work we investigate the interplay between network dismantling and epidemic spread rate as a proxy for the imposition of travel restrictions to control disease spread. For network dismantling we focus on the weighted and unweighted forms of metrics that capture the topological and informational structure of the network. Our results indicate that there is benefit to a directed approach to imposing travel restrictions, but we identify that more detailed models of the transport network are necessary for definitive results.

## Introduction and motivation

### Background

Since December 2019 the world has been adjusting to life with COVID-19, with the first outbreak being reported in Wuhan in China (Wu et al. 2020). COVID-19 is a disease caused by the highly contagious SARS-CoV-2 virus and is characterised by severe respiratory complications and high fatality rates. Societies worldwide are facing up to the first global pandemic since the ‘Spanish Flu’ outbreak of 1918; a so-called once in a century event. What is very evident from available data is that the epidemic will very sadly continue to claim lives until a cheap and simple treatment is available or the recently developed vaccines are distributed widely. At the time of writing worldwide fatalities are approaching four million individuals (Gardner 2020), and with the arrival of the so-called delta variant the ‘third wave’ is well underway as we head into the northern hemisphere summer. Public policy towards control of this disease has mostly focused upon social-distancing measures to break the chain of infection, and more recently alongside the roll-out of novel new vaccines. Social distancing as a policy is based upon the idea that SARS-CoV-2 is spread via person to person contact, and by reducing social mixing the epidemic will be significantly slowed. This approach is based upon the well understood models of network epidemiology which in turn relies upon a contact graph (Pastor-Satorras and Vespignani 2001; Newman 2002; Kiss et al. 2017) over which the network spreads.

Interruption of this network of social contacts via a lockdown has serious economic consequences. In the initial phases of the pandemic the consequences of the lockdown included a drop of $87.5\%$ in airline traffic in China accompanied with a drop of $21.2\%$ in retail sales (Malden and Stephens 2020), and a precipitous contraction of $20.4\%$ in the Gross Domestic Product of the United Kingdom (Scruton 2020) as examples. Although economies recovered moderately in the summer of 2020, and again in the spring of 2021, economic activity is still well below normal.

The purpose of this paper is to investigate how the two deleterious consequences of the pandemic may be balanced. On the one hand it is unrealistic for economies to remain locked down, and on the other it is vital to control the speed of the epidemic to minimize fatalities. At the heart of all economies are transport networks, and the capacity of transport networks is an indicator of economic activity. We focus upon the airline transport network as detailed data is available on routes (OAG Yearly Historic Flight Schedules, Open Data from OurAirports)﻿, capacity and timetables, although the methods and analysis are applicable to any transport network. It is also interesting to note that certain *Agent Based Models* of epidemic spread focus upon airports as the principle point of ingress of a pathogen (Cliff et al. 2018), and so controlling the spread through the airport network would significantly reduce community spread inside of geographically isolated nations such as Australia.

The role of the airline transport network in epidemic spread has been studied previously (Colizza et al. 2006; Balcan et al. 2010; Bajardi et al. 2011), although the results do not provide a clear template as to how the network could be restricted to slow down epidemic spread. In particular the Bajardi et al. study noted that even with a 40% reduction in airline traffic between the US and Mexico the epidemic of H1N1 did finally break through. The 40% reduction was achieved by the effective isolation of Mexico into quarantine, by severely restricting direct international travel, but of course there are many ways to getting to and from a country that do not involve a direct flight which may explain the epidemic breakthrough. The transport network, considered in isolation, is a graph over which the epidemic can be modelled as a spreading phenomenon. The removal of links from this graph will eventually break it down into disconnected components, a process known as ‘dismantling’ (Tian et al. 2017; Wandelt et al. 2018). Clearly when a transport network is dismantled it is not possible for an epidemic to traverse from one disconnected component to another. However, this is a rather dramatic way to contain an infection and the central aim of our work is to see how possible it is to balance route carrying capacity reduction, as the route network is dismantled, with the reduction of the speed of epidemic spread. Although many schemes for dismantling have been studied for efficacy in the speed with which a network may be deconstructed (Requião da Cunha et al. 2015; Zdeborová et al. 2016; Braunstein et al. 2016; Morone and Makse 2015; Wandelt et al. 2018), the majority of the networks studied are unweighted. To truly model capacity we need to introduce the concept that all links are not equal, and this can best be represented by weighting the links with a capacity metric. With this addition we can assess the dismantling approaches with respect to the reduction in total carrying capacity of the network, which we can then balance against a simple model of epidemic spread on that network. Together with a metric of graph entropy that measures the information content of the graph on a node by node basis, and known as *Vertex Entropy* (VE) (Tee et al. 2017, 2018), we compare a number of dismantling schema on capacity and epidemic spread. In the cited studies of network dismantling the leading metric, as measured by the ‘Robustness’ metric is the *Betweenness Centrality* (BC) (Wandelt et al. 2018), which identifies nodes that lie on the most shortest paths between node pairs in the network. The robustness metric essentially computes how fast the network is dismantled by measuring the reduction in the size of the *Giant Component* (GC), and so this is perhaps unsurprising. Betweenness Centrality is expensive to compute and misses some of the subtleties of inter-connectivity, which in our study is the availability of indirect flights between destinations. Vertex Entropy is much cheaper to compute than BC and has been seen to act as a good approximation to it in other applications (Tee et al. 2017). Although VE is closely correlated with centrality measures, it does not exclusively identify the hubs or connectivity ‘pinch points’ in the network, but will also identify nodes on highly critical paths through a network that are not necessarily the shortest ones. We speculate that these nodes represent important pathways for spreading phenomena, but are not necessarily high capacity routes in the network, and seek to verify this numerically in the “Simulations and results” section. The principle contribution of this work is that VE is at least as effective as BC in preserving capacity whilst slowing epidemic spread, it also has the advantages of being easier to compute, and a simpler and more natural extension to weighted networks. This extension to weighted networks of the version of VE that we consider here is novel and not previously studied, and we outline the theoretical treatment in the “Theoretical considerations” section.

The answer to the question that we pose, rests upon the relationship between the capacity of a transport network and the spread of an epidemic on the same network, as modelled by the site percolation model described in “Materials and methods”. Specifically, we are able to compare different schema for the reduction of capacity of the network on their effect of the rate of spread of the epidemic as measured by the effective transmission rate, $R_e$. We are careful to stress that this is not the basic reproduction rate $R_0$, as the primary purpose of our model is to experiment with network restriction, not provide robust predictions of epidemic spread. Indeed, producing an accurate model of the epidemic spread on the airline transport network is not the primary goal of this work. Building such a model would be an extremely complex undertaking, and, for the purposes of the question we address in this work, unnecessary. For our requirements, a consistent model of epidemic spread, dependent upon the network structure, is more important and allows us to compare the various network dismantling schemes. The approximate model yields the value $R_e$, as described above, which, although not in any way an indicator of $R_0$, allows this comparison in a quantitative manner. Nevertheless, we are able to show that there is a significant difference between random closure of airports and routes and a selective method that uses graph properties of the network to select airports and routes to close.

In analyzing the schema for route limitation in the transport network we focus upon the network structure specifically targeting nodes. It is well known that real world networks, particularly those possessing the scale-free property have non trivial behavior as links are progressively removed (Albert et al. 2000). In particular the collapse of the Giant Component (GC) exhibits a second-order phase transition (Xiao et al. 2015), which although originally studied in scale-free graphs, is in principle present in all graphs with a long tail degree distribution. The random removal of links tends not to provoke functional failures of a network (as measured by the number of nodes that become unreachable), but targeted removal of hubs (nodes with a large degree or number of links) can provoke failure very quickly, essentially accelerating the change of ‘phase’ from highly connected to disconnected. It is this critical behavior under node removal that provides the motivation for our approach, as the connectivity of a network has a profound impact on the speed with which an epidemic can spread, as modeled by a random walk of infectious individuals upon it. In essence, if velocity of the epidemic spread reduces linearly with the number of nodes removed, it is possible that prior to the phase transition and collapse of the GC that the epidemic will be retarded whilst the capacity is less affected.

Indeed the relationship between $R_e$ and network capacity is complex and non-linear. In the cases we examine, we show that for a drop in $R_e$ of 0.15, the capacity of some of the underlying networks can be 50% greater than for the random removal case. We believe this justifies the principle of partial network restriction for epidemic moderation.

It is possible to refine further the methods used to model the spread of SARS-CoV-2 in a much more granular fashion. We believe these results provide a motivation to produce those more detailed models and that those models have the potential to form the basis of an alternative approach to managing this and future pandemics, rather than repeated and complete closure of transport infrastructure.

### Outline of this paper

We begin in “Theoretical considerations” with an overview of the necessary concepts of network epidemic models and vertex entropy in which to frame the experiments we undertake. We describe the simulation and experimentation in the “Materials and methods” section, outlining the construction of both our epidemic spreading model and also the route restriction methodology. In “Simulations and results” we discuss the results obtained and draw to a close in the “Conclusions” section, where we include an outlook for further work.

## Theoretical considerations

### Notation

Throughout this paper we follow the conventional notation for graph theory defined in standard texts (Bollobás 1998). A graph is a collection of vertices *V* and the edges, ${E\! \subset \! V\! \times \! V}$, that exist between those vertices, which we write as *G*(*V*, *E*). An edge, represented by $e_{ij}$, denotes a link between vertices $v_i$ and $v_j$. We assume that the graph is simple and undirected. Later in the analysis we will have cause to examine weighted graphs, which we denote by *G*(*V*, *E*, *w*), where *w* represents the weighted adjacency matrix. We define *w*, such that the weight of an edge, is defined as ${w_{ij}}$, where ${w_{ij}=0}$ if there is no edge between $v_i$ and $v_j$. Also, as the graphs we consider are simple and undirected, $w_{ij}$ is symmetric and $w_{ii}=0$

For both weighted and unweighted graphs, we can form an induced subgraph by considering a subset of its vertices ${S\! \subset \! V}$, and the subset of edges $e_{ij} \in E$, where both vertices $v_i,v_j$ are in *S*.

### Network epidemic models

As was noted in the introduction, infections require a physical means of transferring from an infectious individual to a susceptible one. Some diseases, such as sexually transmitted ones, require physical contact for transmission to occur, whereas airborne infections, believed to include SARS-CoV-2, only require proximity. Whatever the biological mechanisms, at the core of epidemic spread is a network of contacts. A contact network represents individuals (or places) as nodes, and the links represent a contact along which transmission can occur. The disease then proceeds by transmission along the links usually governed by a transmission probability. The structure of the network has an important role on the progression of the network (Bell et al. 2020; Newman 2002), and provides the starting point for the strategy we investigate in this paper.

In particular, many real-world networks are known to possess the ‘small world’ property (Watts and Strogatz 1998), involving the presence of hubs that create short cuts in the network and dramatically reduce the graph diameter. In principle, fewer network hops are required on average to reach a node, with obvious implications for epidemic spread. This property can be replicated with networks that are generated by the various forms of preferential attachment (Barabási 2016; Albert and Barabási 2002), which produce a scale-free degree distribution. It is an often-cited claim that real-world networks have a power-law degree distribution where ${p(k)\propto k^{-\alpha }}$; with values of $\alpha$ typically in the range ${2.0\text {--}3.0}$ and *p*(*k*) being the probability of a randomly chosen node having degree *k*. This claim has been much disputed (Broido and Clauset 2019), and indeed contact networks may not be scale free (Vanhems et al. 2013). It has been hypothesized that transport networks exhibit a reasonably strong scale freedom (Sridhar and Sheth 2008), however, our analysis of the airline network does not agree with this conclusion. Using the approach outlined in Broido and Clauset (2019), we performed a goodness-of-fit test for the airline network degree distribution against the power law, truncated power law, log-normal, and exponential distributions. Our analysis showed the closest fit to be a truncated power law. This result is intriguing, because although the airline network may not itself be scale free, it does exhibit similar resilience behavior. Of course, networks have many properties beyond degree distribution, each with potentially different scales. In fact Zhou et al. (2020) define a new property, *degree-degree distance*, whose distribution has been shown to exhibit a better power-law fit than degree. The key point however, is that the presence of hubs, connecting distant parts of the network, could underlie the collapse of the giant component upon targeted node removal.

In our work we adapt the percolation approach to modeling epidemic spread (Moore and Newman 2000; Newman 2002). The percolation model depends upon each link in a contact network permitting transmission of the disease according to a probability *T* called the transmissibility. Effectively, one starts with one infected node and then as a path of infection is traversed the link is marked as ‘occupied’ and the component of the graph connected by such links emerges as an *infected cluster*.

The transmissibility governs the size of the infected cluster and is dependant upon the length of time an individual is infectious $\tau$, and the rate $\beta$ at which an individual infects one of its contacts. Providing $\beta$ is independent of time, in terms of these parameters its value is $T=1-e^{- \beta \tau }$. As this probability varies from 0.0 to 1.0 the size of the infected cluster does not vary smoothly, but in general transitions to a large fraction (${\text {say}>50\%}$) at a distinct value of *T*. That is, the epidemic undergoes a phase transition (Dorogovtsev et al. 2008), and this critical behavior is a function of the control parameter *T*. Below a critical value $T_c$ the size of the infected cluster is very small and the epidemic not widespread. Above $T_c$ the spread affects a finite fraction of the population.

We describe in “Materials and methods” how we apply transmissibility and percolation to our simulation, but at coarse scale it is not meaningful, in a network carrying millions of passengers, to model every interaction between infectious and susceptible passengers. Instead, we use the concept of a contact probability of transmission to determine the number of infected passengers that exit a flight dependant upon the capacity of the aircraft. This is a key simplification (and vulnerability) of our model, and in future work we intend to expand our model to take account in a more granular fashion the person to person interactions of our infected individuals.

This detail notwithstanding, it is clear that the value of $\beta$ plays an important role whether the epidemic spreads at all on our toy model. We assume for the purposes of exploring the effectiveness of our route closure selection schema a value of $\beta$ that will lead to an endemic spread of the infection.

A key metric in our analyses is the effective transmission rate, $R_e$. As we stated in the “Introduction and Motivation” section, $R_e$ is not the basic reproduction rate, generally denoted as $R_0$, and which is the number of secondary infections created by a single infected individual. We assume that in the early stages of the epidemic that the growth of the epidemic is exponential in time, consistent with *Susceptible-Infected-Recovered* (SIR) compartmental models of epidemics (Bell et al. 2020). Early in the epidemic if *I*(*t*) is the proportion of infected individuals as a function of time, then $I(t) \propto e^{R_0 t}$ (Kiss et al. 2017). This result is only valid when *I*(*t*) and *R*(*t*) are small relative to the population, and can only be asserted early in the progress of the pandemic. Our computation of $R_e$ is taken by making the assumption that ${I(t) \propto e^{R_e t}}$ and extracting $R_e$ by fitting our epidemic spread to this relationship. We specifically do not intend this *ansatz* to imply any direct relationship between $R_e$ and $R_0$, but instead justify the use of $R_e$ as a measure of epidemic spread for our model.

### Vertex measures of graph entropy

The concept of graph entropy was introduced by Körner 1986 and Simonyi 1995. Since then many approaches (Passerini and Severini 2008; Bianconi 2007, 2009) have emerged to analyze and quantify the information encoded in the structure of a graph, in particular how the potentially vast configuration space of graphs that share common features (such as degree distribution) effectively ‘hide’ information and therefore have entropy. In essence, graph entropy measures the complexity of a graph but it is neither easy nor efficient to compute. For example, the original definition of Körner relies upon determining the stable sets of a graph, a well known NP-complete problem. For practical purposes it would be ideal if an approximate vertex level measure of entropy were available.

Vertex Entropy (Tee et al. 2017) is one such approach. Initially defined on unweighted, simple graphs, VE is based upon a formalism for vertex level entropy first introduced by Dehmer (2008), and Dehmer and Mowshowitz (2011). Dehmer utilized the concept of a *local functional* for a vertex, which can be scoped to calculate values for every vertex based upon the local topology of the graph. The degree of locality in the treatment is controlled by using the concept of the *j-sphere*, $S^{j}$, in the graph, centered at a given vertex. For clarity, in the following definition a superscript indicates the order of the *j*-sphere whereas subscripts run over the members of the vertex set of the graph.

The methodology of Dehmer’s original definition relied upon subsets of vertices of a fixed distance from a given vertex $v_{i}$, where distance ${d(v_{i},v_{j})}$ is the fewest number of edges in a walk from $v_i$ to $v_j$. This definition excluded the vertex $v_{i}$, and other interior nodes for ${j \ge 1}$, but this introduces problematic zeroes when we introduce the clustering coefficient. Accordingly, in Tee et al. (2017), we extended the definition to include the vertex $v_{i}$ as part of the set. The definition of $S^{j}$ is then modified as follows. For a graph *G*(*V*, *E*), we define for a vertex ${v_{i} \in V}$, the *j*-sphere centered on $v_{i}$ as:

1 $$\begin{aligned} S^{j}_{i}= \{ v_{k} \in V \vert d(v_{i},v_{k}) \le j, j \ge 1 \} \cup \{ v_{i} \} \end{aligned}$$

and for convenience we also define the related *j-edges*, $E^{j}_{i}$ as

2 $$\begin{aligned} E^{j}_{i}=\{ e_{kl} \in E \vert v_{k},v_{l} \in S^{j}_{i} \}\text {,} \end{aligned}$$

where $e_{kl}$ represents an edge between vertices $v_k$ and $v_l$.

The concept of *j*-spheres is a convenient formalism to capture locality in the graph and by breaking a large graph into *j*-spheres, we can progressively examine complex combinatorial quantities such as graph entropy on increasingly larger subsets of the graph. We proceed by equipping each $S^{j}_{i}$ with a positive, real-valued function ${{f_{i} : v_{i} \in S^{j}_{i} \rightarrow {\mathbb {R}}^{+}}}$. This function is intended to be dependent upon properties of the nodes that are members of the *j*-sphere, such as their degree, number of cycles and so on, and therefore capture the localized structural properties of the graph. We can then construct a proper probability function for each vertex,

3 $$\begin{aligned} p_{i}=\frac{f_{i}}{\sum _{ v_{k} \in S_{i}^{j} } f_{k}} \text {,} \end{aligned}$$

which naturally satisfies $\sum _{i} p_{i} = 1$. These functions are then used to construct entropy measures in direct analogy to Shannon entropy as follows:

4 $$\begin{aligned} H(v_{i}) = - p_{i} \log _{2} p_{i}\text {.} \end{aligned}$$

A basic form of vertex functional can be chosen to be $f_i=k_i$. The probability in this instance, as defined by Eq. (3), represents the probability of a randomly chosen edge being incident upon vertex $v_i$. This choice gives the first definition of a VE,

5 $$\begin{aligned} H_k(v_{i})=\frac{k_{i} }{2 |E |} \log _{2} \left( \frac{ 2 |E |}{k_{i} } \right) \text {,} \end{aligned}$$

where $|E |$ is the number of edges in the graph, recalling that ${\sum _i k_i = 2 |E |}$. The definition of VE was analyzed in Tee et al. (2017) and termed *Fractional Degree Entropy*.

The influence of a vertex within any graph is likely to be related to how it is connected into that graph, not simply by how many edges are incident upon it. To capture this local structure we consider the concept of vertex clustering first introduced in Watts and Strogatz (1998) as a normalizing factor in our definition of a vertex entropy, such that:

6 $$\begin{aligned} H^{\prime }(v_{i}) = \frac{ H(v_{i}) }{ C_{i}^{j} }\text {,} \end{aligned}$$

where $C_{i}^{j}$ represents a local clustering coefficient for the *j*-sphere of interest. The normal definition for the clustering coefficient given in Watts and Strogatz (1998) introduces numerical difficulties for triangle-free graphs. In accordance with the definitions of ${S_i^j}$ and ${E_i^j}$ in (1) and (2) respectively, Tee et al. (2017) modifies the clustering coefficient to include edges incident on $v_i$. We define $C_{i}^{j}$ in terms of the number of edges in a *j*-sphere edge set $|E^{j}_{i} |$, (2), as,

7 $$\begin{aligned} C_{i}^{j} = \frac{ 2 |E_{i}^{j} |}{ k_{i} ( k_{i} + 1 ) } \text {.} \end{aligned}$$

Finally, and for completeness, *Normalized Fractional Degree Entropy* is defined by:

8 $$\begin{aligned} H_{k}^{\prime }(v_{i}) = \frac{ k_{i}^{2}( k_{ i } + 1 ) }{ 4 |E_{i}^{j} ||E |} \log _{2} \left( \frac{ 2 |E |}{k_{i} } \right) \text {.} \end{aligned}$$

### Vertex entropy for weighted graphs

The analysis above is conducted on simple, undirected and crucially unweighted graphs. This last restriction will be problematic for our simulation work, so accordingly, and using the nomenclature defined in the “Notation” subsection of “Theoretical considerations”, we will now introduce an extension to Vertex Entropy for a weighted graph *G*(*V*, *E*, *w*) on ${N=\left| V\right| }$ vertices. As we are still dealing with undirected graphs, we can, instead of using the number of edges incident on a vertex, compute a weighted degree, $K_i$, such that

9 $$\begin{aligned} K_{i} = \sum \limits _{ j = 0 }^{ j < N } w_{ij} \text {.} \end{aligned}$$

To produce a valid probability we need the sum of this weighted degree as the denominator in expressions analogous to Eq. (3). We know that for the unweighted case ${\sum _i k_i = 2 |E |}$, consequently and for a fixed set of weights, we can also write:

10 $$\begin{aligned} \sum \limits _i K_i = W \times 2 |E |\text {,} \end{aligned}$$

where W is a constant. We retain the factor of 2 in Eq. (10) to emphasize the proportionality ﻿of $\sum \limits _i K_i$ to the sum of unweighted degree, which is 2|*E*|. We could of course absorb this factor of 2 into *W*, but retention highlights the relationship ﻿between $\sum \limits _i K_i$ and﻿ ﻿$\sum \limits _i k_i$. It suffices now to normalize the weighted degree, by dividing by *W*, so that ${K_i^\prime =K_i/W}$, and we can define our vertex functional $p_{i}^{w}$, as follows:

11 $$\begin{aligned} p_{i}^{w} = \frac{ K^\prime _{ i }}{ 2 |E |} \end{aligned}$$

with identically,

12 $$\begin{aligned} \sum \limits _{i} p_{i}^{w} = 1 \text {.} \end{aligned}$$

We now use the the well defined probability ${p_{i} = K^\prime _{i}/2 |E |}$ to define our *Weighted Fractional Degree Entropy* as:

13 $$\begin{aligned} H_K^\prime (v_{i})=\frac{K^\prime _{i} }{2 |E |} \log _{2} \left( \frac{ 2 |E |}{K^\prime _{i} } \right) \text {,} \end{aligned}$$

with its normalized counterpart given by:

14 $$\begin{aligned} H_{K^\prime }^{\prime }(v_{i})= \frac{ H_{K^\prime }(v_{i}) }{ C_{i}^{j} }\text {,} \end{aligned}$$

and hence, for completeness,

15 $$\begin{aligned} H_{K^\prime }^{\prime }(v_{i})= \frac{1}{C_{i}^{j}} \frac{K^\prime _{i} }{2 |E |} \log _{2} \left( \frac{ 2 |E |}{K^\prime _{i} } \right) \text {,} \end{aligned}$$

where $C_{i}^{j}$ is the unweighted, local clustering coefficient given by Eq. (7).

### Weighted betweenness centrality

A key metric we use to investigate the dismantling of the airline route network is betweenness centrality. A measure that metricates the number of shortest paths between all pairs of nodes in a graph that pass through a given node. Many algorithms exist to compute BC on unweighted and weighted graphs, with the Brandes algorithm (Brandes 2001) representing the most efficient.

To implement the weighted version of BC in our model however requires some care. Typically you would want the weight in the computation to represent ‘distance’, with larger values indicating a longer link so that the BC value for a node captures the ‘flow’ distance through the graph. The weights that we utilize however act in the opposite direction, in the sense that a higher passenger carrying capacity represents a link that is more likely to transmit infection. As such we need to transform the weights into a distance to compute weighted BC, using a decreasing function in the link weight $w_{ij}$. It is important that any parameters in this function do not affect the ranking of the nodes by weighted BC and we have conducted experimentation to determine an appropriate function. If $D_{ij}$ is the distance weights for the computation of weighted BC, $w_{max}$ the maximum weight of the graph, we define the following transform,

16 $$\begin{aligned} D_{ij} = 1-\frac{w_{ij}}{w_{max}} + \epsilon \text {.} \end{aligned}$$

In this definition $\epsilon$ a small positive parameter necessary to avoid zero values of $D_{ij}$ that cause instability in the computation. Experimentally we determined that a value of $\epsilon =10^{-6}$ was effective at avoiding the zero length instabilities whilst not affecting the ranking of graph nodes by weighted BC.

## Materials and methods

### Data source and data manipulation

The airline route graph used in our analyses was constructed using flight data from the Official Aviation Guide and airport data from ourairports.com. Each entry within the flight database includes departure and arrival airports, airline and flight number, flight duration, plane capacity, code-share data, annual flight schedule etc. Best efforts were made to remove duplicate entries arising from code-sharing and multi-leg flights.

We define a route as any pair of airports between which there is at least one flight. For each route we aggregate flight details from across the year to give metrics such as flight distance, flights per day, passengers per day, and flight capacity.

More formally, we define our airline route graph as an undirected, weighted graph, *G*(*V*, *E*, *w*), where *V* is the set of airports, *E* the set of routes, and *w* the matrix of weights for individual routes such as its capacity in passengers.

### Overall objective

Using this weighted graph we simulate the spread of a disease by modelling a random walk of infectious individuals across the route network. As nodes are visited we mark the site as infected and continue our random walk, propagating the epidemic by using a coarse grained approach to infection using the concept of transmissibility.

The coarse graining is obtained by categorizing the routes by the average capacity of flights operating between the airports. For each arriving flight that contains an infectious individual we use a probabalistic model to assess any onward infection. In the event that onward transmission has occurred, we assume that the infected individuals become infectious immediately, and hence they become transmitters of the virus on the next step of the random walk. This of course is not a realistic assumption for a true model of epidemic spread, not least because the serial interval, being the time between becoming infected and infecting another person, is estimated to be ${3\text {--}7}$ days for SARS-CoV-2 (Park et al. 2020). Our justification is that we are effectively ‘compressing’ time by making this assumption, and we are principally interested in the overall effect of network changes on the spreading phenomenon, not detailed predictions for the actual spread of the disease—that would require a much more detailed approach such as an agent based model (Cliff et al. 2018). We compute averages over multiple runs of the simulations, each of which are terminated when $80\%$ of the nodes become infected and then historically compute the $R_e$ value of the infection. The computation of $R_e$ is undertaken by optimizing a fit for the early part of the infection using an exponential spread model and extracting the reproduction rate.

### Simulation detail

Every random walk starts at a random node in the graph. The onward step at every stage of every walk is chosen using a probability weighted by the number of passengers travelling along each route from the current location. Intuitively, this describes the case of a passenger randomly picking a single ticket from the set of all tickets available across all flights for all destinations available from that airport.

Our analyses consider seven approaches to restricting the size of the network. The first regime is based upon random selection. A further six use targeted approaches based upon the local structure of the graph: node degree; Vertex Entropy, Eq. (8); Betweenness Centrality; weighted degree, Eq. (9); weighted Vertex Entropy, Eq. (15); and weighted Betweenness Centrality. We use the number of passengers along each route as the basis for the weighting criteria. For degree and Vertex Entropy we use this quantity directly, and apply the transformation of Eq. (16) for Betweenness Centrality. For random removal, and in order to give a more realistic simulation, we consider only those airports categorized as *large* by ourairports.com.

The number of onward infections is chosen in a two-stage process. Our simulation requires discrete walkers. To facilitate this, we map a nominal value for $R_e$ (in this case 1.5), onto a discrete probability density function and randomly choose the number of onward infected walkers from that distribution. The probability density function is a truncated normal distribution with a mean of $R_e$, a standard deviation of ${R_e/2}$, zero as the lower limit of the distribution, and no upper limit. Our choice here of a random distribution of newly infected onward walkers reflects the lack of detail in the model regarding the behavior of the infection during the flight taken by the original infected passengers. To accurately reflect the number of onward walkers would involve detailed modeling of the transmission of the disease in an airplane, taking into account occupancy, seat layout, plane ventilation, social distancing measures at airports and many other factors. However, the number of individuals infected during the flight will also be driven by the basic reproduction number, with a higher value resulting in more infections. We make the assumption that the number of new infected travellers will depend upon a collection of independent random factors, each of which will have a potentially complex relationship to $R_e$. We then appeal to the central limit theorem to assert that the number of onward walkers can be modelled as a normal distribution, around $R_e$, and choose the variance such that $95\%$ of the onward walkers laying between 0 and $2R_e$. In order to introduce the effect of flight capacity characteristics into the transmission model we factor this value based on the average capacity of the planes serving the route. We use a factor of 2 for medium aircraft ($150\text {--}300$ passengers) and a factor of 3 for larger aircraft. The values chosen for these factor is somewhat arbitrary and so a limited number of different values were assessed. We observed some variation in the absolute values of $R_e$, as would be expected, given that the factors directly impact the number of onwards transmissions. However, the qualitative behaviour and relative performance of the different removal regimes were largely unchanged. We remind the reader that the purpose of our epidemic model is to have a qualitative comparison of different dismantling regimes on epidemic spread rates, rather than extracting detailed epidemic metrics such as $R_0$ or final epidemic size. The faithfulness of the model to the detail of an actual epidemic is in this way less important than having a consistent experimental framework. Regardless of these observations we do acknowledge the limitations of the current approach and consequently consider an improved model of transmission dynamics to be a key part of our future research.

To cover the different node removal regimes we adopt two different approaches to the random walks. The common elements of both are: nodes are removed from the graph until its GC reduces to $80\%$ of its original size; and each random walk terminates once $80\%$ of the nodes in the original graph have become infected. The reasons for our choice of ${80\%}$ for the GC size and the infected node thresholds are two-fold. In order to minimize any potential bias in our simulations introduced by network size, we wanted to ensure that we retained as much of the original GC as possible during the network dismantling process. However, and contrary to this first objective, we also needed to ensure that the capacity of the dismantled network could be both heavily restricted and that its ongoing rate of decay was both small and reducing. Preliminary simulations showed that both of these criteria could be met with an ${80\%}$ threshold on the size of the reduced GC.

For the targeted approaches we remove nodes in descending order of the value of the removal criterion i.e. degree, VE, BC, weighted degree, weighted VE, or weighted BC, based on the initial route graph with no nodes removed. For each node that is removed we conduct 100 random walks.

The random removal regime is more complex as there are two sources of randomness: the nodes to be removed from the graph, and the random walks taken upon those graphs.

To create each randomly reduced graph, we remove an independently and randomly chosen set of vertices from the original graph. The initial set of removed vertices has a cardinality of one. The cardinality is repeatedly incremented by one until the GC of the reduced graph falls below the size threshold as described above. For each cardinality of the removed vertex set we create 20 different sets of vertices and hence 20 different graphs. Upon each randomly reduced graph we conduct 50 random walks. Consequently, for each value of the cardinality of the removed vertex set, we conduct a total of 1000 random walks, specifically 50 walks on each of the 20 randomly reduced graphs.

We acknowledge the different number of “repeats” used for each cardinality of the removed vertex sets for the different removal regimes, specifically, 100 repeats in the targeted removal cases and 1000 repeats for the random removal case. Preliminary simulations showed that the results generally converged within ${50\text {--}60}$ repeats for targeted removal and within ${400\text {--}600}$ repeats for random removal. We attribute the difference in convergence rate to the “double randomness” of the random removal case.

Removing vertices from the graph is analagous to ‘closing’ airports and all associated routes. As we do this we compute the passenger carrying capacity of the remaining network as measured by *passenger-kilometers* (pkm). Our experiment is to investigate the relationship between capacity and $R_e$ using these different schema, with the objective of identifying improved methods for network restriction whilst preserving the network’s capacity.

## Simulations and results

In the following section we present the results of our simulations and highlight the key observations. Our primary objective is to study the impact of seven different node removal regimes on epidemic propagation, specifically: random removal of nodes representing large airports, targeted removal based on the weighted and unweighted forms of degree, vertex entropy, and betweenness centrality. All other free-parameters and simulation techniques such as the transmission mechanism, random walk stopping criteria, route selection, etc., remained the same as described in “Materials and methods”.

**Fig. 1:**
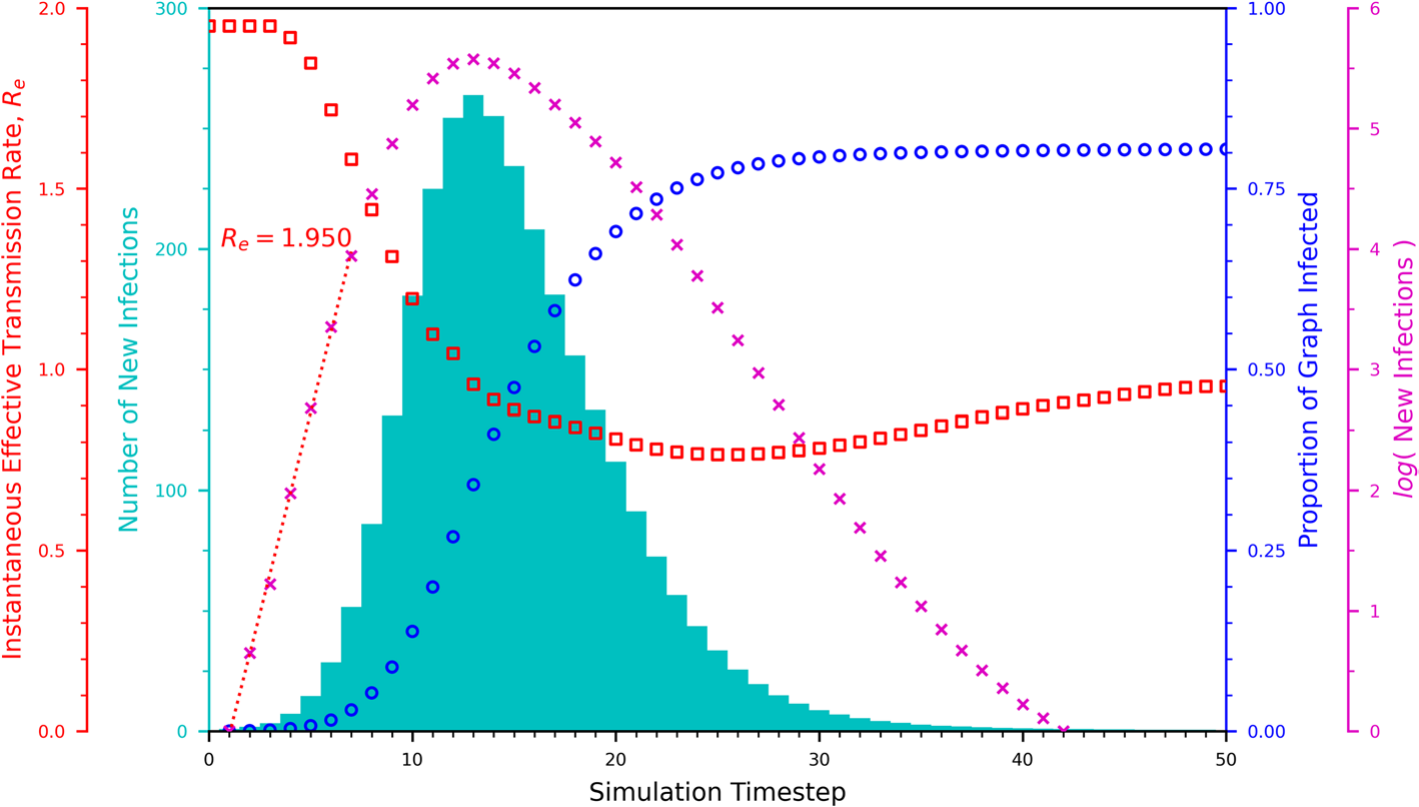
Aggregated metrics for the spread of an epidemic on the complete airline route graph. The metrics presented are the number of new infections per time-step, the total proportion of the GC that has become infected and the effective transmission rate. The results are the average of 1000 repeats of a random walk, each walk starting at a randomly chosen node. The results exhibit the same characteristics of the well-know SIR compartmental model of epidemic spread

We begin the presentation of our results by demonstrating the estimation of $R_e$. The data shown in Fig. 1, is representative of all of our simulations, and in this case represents the aggregation of data across multiple random walks over the complete airline route graph with no nodes removed. The number of random walks used to extract aggregated metrics, such as $R_e$, and capacity etc., depends upon the node removal regime under consideration, as described in the “Simulation Detail” subsection of “Materials and methods”. For the case show in Fig. 1 the results have been aggregated over 1000 random walks.

The dependant variable in each simulation is the number of new infections per time-step. In the early stages of the epidemic, infections grow exponentially, peaking in this case, at time-step 13. The behaviour of the new infection count, and the cumulative proportion of the graph that has become infected, are characteristic of the SIR model of epidemic spread (Kermack and McKendrick 1927).

We estimate the instantaneous value of $R_e$ at each time-step by finding the line of best fit to the $\log$ of the new infection count using a simple least-squares regression centred on the time-step of interest. The instantaneous rate reduces from its peak in the early part of the epidemic, dropping below 1.0 after the peak of new infections. As the epidemic continues, $R_e$ reaches a minimum of approximately 0.75, before asymptotically increasing to a value of approximately 0.95 as the graph-infection threshold of $80\%$ is approached.

Of course, for an epidemic, the $R_e$ metric characterizes the exponential growth of the infection, and has validity in the early phase of the epidemic spread. As can be seen from Fig. 1, there is a plateau where the growth in infected individuals is strongly exponential at $R_e=1.950$, and it is this value that we record for this experiment. To extract the value of $R_e$ for a given experiment, we identify the early plateau and use the corresponding value of $R_e$. The results of each experiment are presented as individual data points on the figures presented later in this section.

**Fig. 2:**
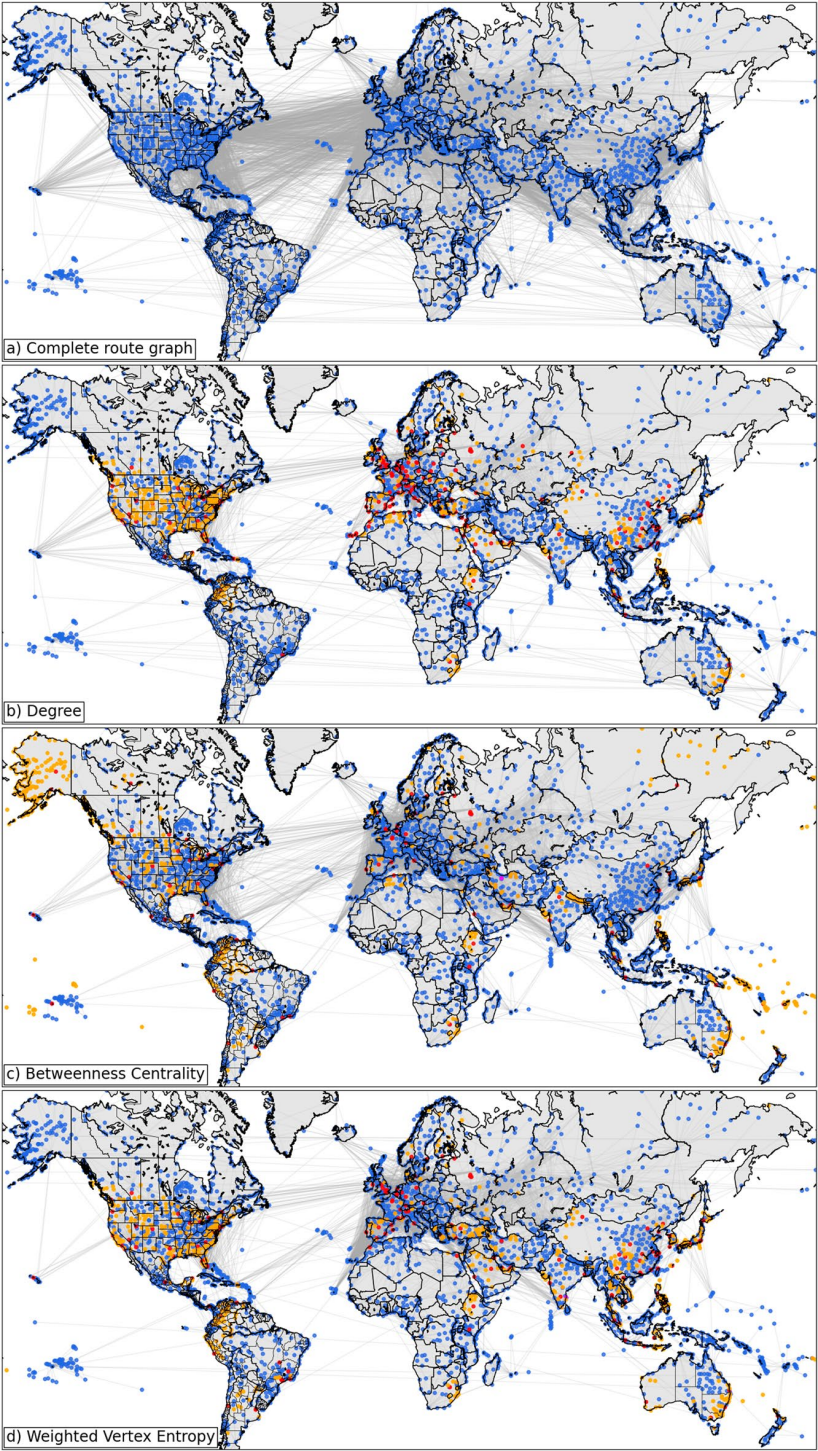
The global airline route maps under different network dismantling regimes. **a** Shows the complete graph with no nodes removed. **b**–**d** Show the route graph when the GC has been reduced to $80\%$ of its original size using degree, betweenness centrality and weighted vertex entropy respectively. Open airports are represented by blue dots, removed airports by red dots and disconnected airports by orange dots. A high resolution version of this figure is available at 10.6084/m9.figshare.14994246

Figure 2 shows the qualitative impact of three different node removal strategies. Figure 2a shows the map of all airports and routes in the global network prior to any node removal. Each dot represents an airport and each line a route. Figure 2b–d show the impact of reducing the size of the original network by $20\%$ using degree, betweenness centrality, and weighted vertex entropy respectively. Red dots represent airports that have been removed from the network, while orange dots indicate airports that have subsequently become disconnected from the core of the graph.

In all removal scenarios the impact on global routes is profound with large reductions in open routes across the world. It is interesting to note that the different removal regimes have impacted different geographical regions. One of the most obvious differences is the reduced volume of open routes between Northern Europe and North America. While all removal regimes cause a substantial reduction in this region, the degree-based removal regime retains the fewest open routes and the BC removal regime the most. As has been previously remarked the airline route graph is intentionally structured around hubs so as to reduce the graphs diameter. This structural feature of the graph would explain the tendency of degree based removal to reduce routes more quickly than say BC. Other regions of note include Alaska and the South Pacific Islands. For BC-based removal, the entirety of Alaska has become disconnected following removal of the key hubs of Anchorage and Fairbanks International airports. Contrast this with the degree and weighted VE-based case where all of the Alaskan airports remain connected. Similarly the BC regime disconnects many of the airports serving the South-Pacific Islands. Comparison of the BC and weighted VE-based regimes shows there is a broad similarity in the retained routes across Africa, with some minor, but noticeable differences across Europe, South America, Western Australia and India. More significant differences can be observed across the Middle-Eastern region and South-East Asia with the weighted VE regime removing many more routes than BC.

The quantitative impact of the different removal strategies on the network metrics when the GC is reduced to $80\%$ of its original size is shown in Table 1. Comparison of the unweighted degree and VE cases with BC-based removal shows that approximately $50\%$ more nodes need to be removed in the VE-based case and in the degree-based case more than twice the number of nodes must be removed to achieve the $20\%$ reduction in the size of the GC. This is consistent with both the objective of VE as an efficiently computable metric to identify single points of failure more accurately than degree and the previously observed effectiveness of BC as a network dismantling metric. However, we highlight that that the objective of this work is to assess the impact of these metrics on restricting epidemic spread rather than their efficacy as network dismantling metrics. We move now to the number of retained edges. For both the degree and VE removal regimes, the number of edges falls, very roughly, by two-thirds with a corresponding, and approximate, $90\%$ collapse in network capacity. The number of retained edges and the retained capacity of the network for the BC removal regime is considerably higher. Capacity falls by approximately $75\%$ and the number of edges by only $40\%$. These results suggest that BC retains higher capacity routes than both the unweighted degree and VE-based removal regimes.

**Table 1:** Network metrics under different node removal strategies when the GC has been reduced to $80\%$ of its original size

Node removal strategy	Removed nodes	Disconnected nodes	Retained nodes	Retained edges	Retained capacity ${\times 10^9\text { pkm}}$
None	0	0	3661	24683	9.74
Degree	191	540	2930	7805	0.80
Vertex entropy	140	592	2929	9774	1.06
Betweenness centrality	91	636	2934	14491	2.01
Weighted degree	165	567	2929	10011	0.65
Weighted vertex entropy	154	576	2931	9938	0.63
Weighted betweenness centrality	85	625	2951	15477	2.40

The results for the weighted variants of degree and VE are closer to each other than for their unweighted forms. Comparison of the weighted degree and weighted VE variants show that weighted VE requires approximately $7\%$ fewer nodes to be removed, however, the number of edges in the resulting graph differs by only $1\%$. This would suggest a higher degree of similarity between the sets of removed nodes in the weighted cases than for the unweighted case, we revisit this observation below. In regard to capacity, the overall reduction is slightly higher at approximately $93\%$. The high-level observations made above regarding the number of removed nodes and retained edges for the unweighted cases also hold for the weighted cases. To achieve the 20% reduction in GC, weighted VE and weighted degree require significantly more nodes to be removed than for weighted BC. And the number of retained edges in the weighted BC case is higher than for weighted degree, weighted VE, and its unweighted counterpart. There is however a key difference when we examine the retained capacity. For weighted VE and weighted degree, the network capacity reduces when compared with the impact of their unweighted forms, a result that may be expected given that the weighted removal strategies use metrics based upon passenger counts. For weighted BC however, we see the opposite, the retained capacity of the network increases by approximately 20% to ${2.40\!\times \!10^9\text { pkm}}$. The impact of this result is two-fold. Firstly it that shows that while the average capacity of a retained route decreases under the weighted degree and weighted VE regimes, it increases under weighted BC. It also reinforces our earlier observation that BC-based regimes retain higher capacity routes than degree and VE-based regimes.

**Fig. 3:**
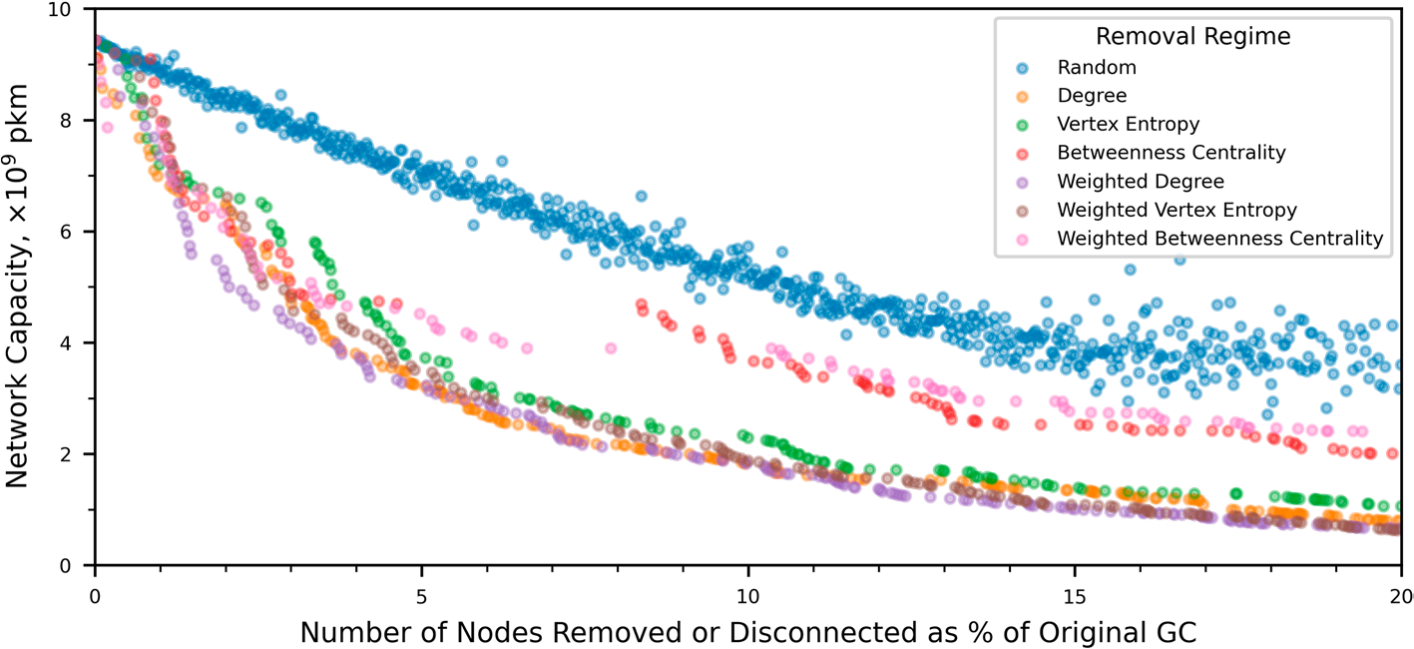
The reduction in passenger carrying capacity of the global airline network as it is dismantled under different node removal regimes. Random node removal causes a smaller reduction than targeted methods. Under targeted removal the network capacity reduces by half when only approximately $3\text {--}4\%$ or the network has been removed or disconnected

The impact of different strategies on network capacity as nodes are removed can be seen in Fig. 3. With a corresponding enumeration of some specific capacities in Table 2. For the case of random removal, capacity initially reduces almost linearly until approximately $15\%$ of the original route graph has been removed. As more nodes are removed and the size of the network reduces further, the rate of reduction in capacity flattens. The degree of scatter in the data also increases at this point, owing to the smaller sample sizes inherent in the random removal process as the graph reduces to $80\%$ of its original size.

**Table 2:** Retained network capacity (${\times 10^9\text { pkm}}$) for different proportions of removed and disconnected nodes under different node removal strategies

Node removal strategy	Proportion of original graph removed or disconnected							
	$2\%$	$4\%$	$6\%$	$8\%$	$10\%$	$15\%$	$20\%$	$25\%$
Degree	6.47	3.80	2.68	2.17	1.82	1.35	0.80	0.51
Vertex entropy	6.66	4.77	3.20	2.59	2.29	1.40	1.06	0.90
Betweenness centrality	6.16	4.76	4.70	4.69	3.68	2.52	2.01	1.66
Weighted degree	5.15	3.75	2.90	2.13	1.82	1.00	0.66	0.44
Weighted vertex entropy	6.62	4.19	3.01	2.42	1.88	1.09	0.62	0.45
Weighted betweenness centrality	6.05	4.65	4.09	3.90	3.90	2.76	2.40	1.87

In the early part of the network dismantling process, specifically up to approximately $5\%$ of the GC being removed or disconnected, all of the targeted removal strategies exhibit similar characteristics. The very steep reduction in capacity for only small reductions in network size is particularly apparent. In fact a reduction in the size of the network of only about ${3\text {--}4\%}$ can reduce its capacity by half. This dramatic reduction is consistent with the well understood resilience and attack tolerance properties of scale free graphs (Albert et al. 2000). As the proportion of removed nodes increases beyond $5\%$, the capacity characteristics for both BC-based removal regimes diverge from the other removal techniques. For these other removal regimes, the rate at which capacity drops is reduced, but in all cases, when $20\%$ of the original graph has been removed, capacities lie in the range ${0.66\text {--}1.06\!\times \!10^9\text { pkm}}$. For unweighted BC there is a distinct and sudden reduction in the size of the GC from approximately ${4.5\%}$ to approximately ${8\%}$ while the capacity of the network remains constants at about ${4.7\!\times \!10^9\text { pkm}}$. Further examination shows that this occurs when Fairbanks International airport is removed, resulting in approximately 150 airports in Alaska becoming disconnected from the GC. These airports serve a very remote part of the world with routes that carry very few passengers. Consequently their removal has a large impact on the size of the GC but virtually no impact on network capacity. A similar feature is seen for weighted BC between GC sizes of approximately ${7\%}$ and ${10.5\%}$. In common with unweighted BC, this is caused by the airports in Alaska becoming disconnected. Another intriguing, but certainly less dramatic feature of Fig. 3, is the small plateau in capacity using the VE removal scheme near $2.5\%$. For this case, and in contrast to the BC regimes, the nodes that get removed are significant international hubs, including Denver and Minneapolis International airports. However, and in addition to their function as international hubs, these airports also serve many small regional airports with routes that carry relatively few passengers. These regional airports exist either as leaf nodes or nodes of very low degree in the network and become disconnected when the hub they connect into is removed. As a result, there is a significant reduction in the size of the GC and a comparatively small reduction in network capacity.

**Fig. 4:**
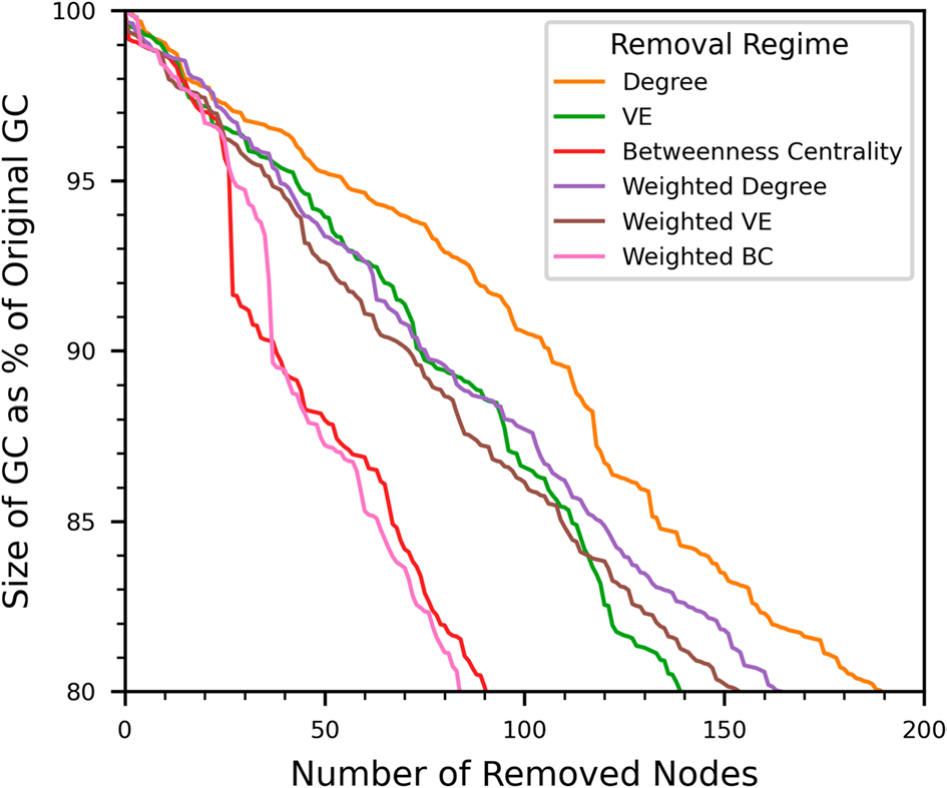
The reduction in the size of the giant component of the airline route graph as the number of removed nodes increases under targeted node removal strategies. Note that the removed node count does not include disconnected nodes. The rate of GC dismantling is fastest for Weighted Betweenness Centrality and slowest for degree. Weighted degree and both forms of Vertex Entropy show similar behaviour with the GC reducing marginally more quickly for unweighted Vertex Entropy

**Fig. 5:**
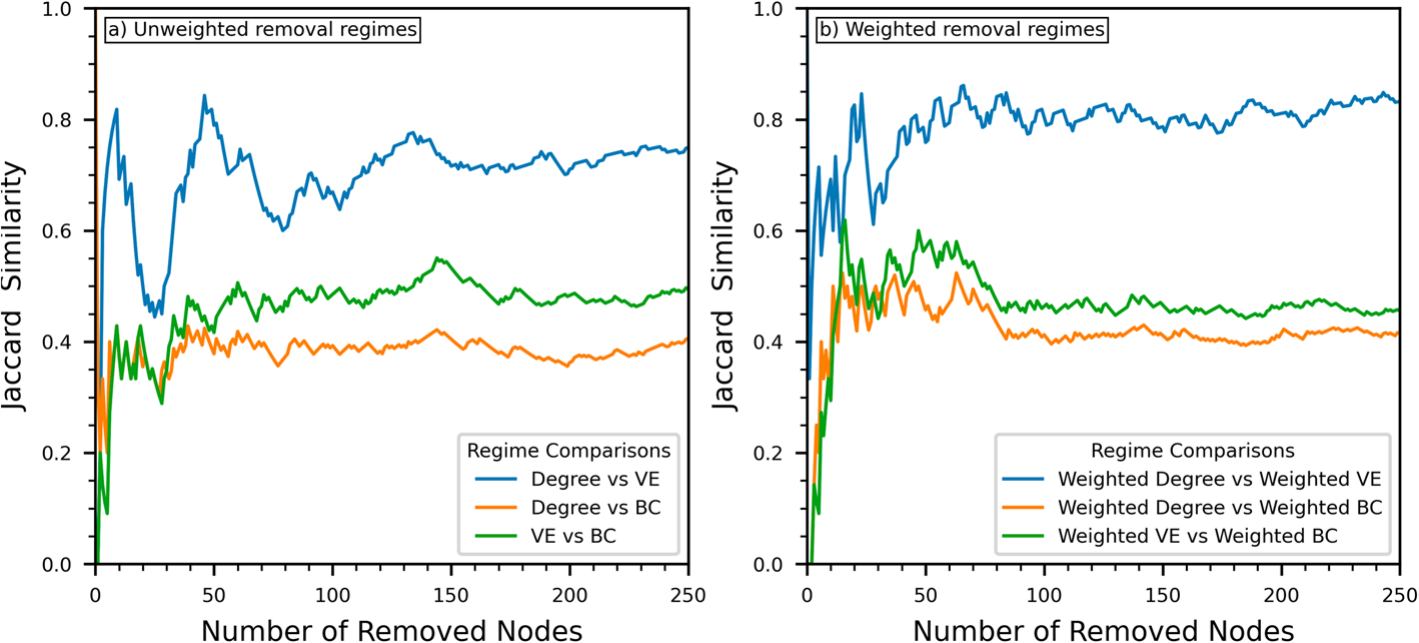
The Jaccard similarity coefficient of the node sets removed from the airline route graph under targeted node removal. **a** Compares the unweighted forms of degree, vertex entropy and betweenness centrality. **b** Compares the weighted forms of degree, vertex entropy and betweenness centrality

We investigate the similarity of the removed node sets for the targeted removal regimes in Fig. 4, which shows how the size of the GC changes as we remove nodes and in Fig. 5, which compares specific node sets using the well-known *Jaccard similarity coefficient* (JC).

Figure 4 shows that the rate at which the GC collapses is similar for the weighted degree and both of the VE strategies. The rate of collapse is slowest for unweighted degree and fastest for weighted BC. For both BC regimes, it is worth noting that without the sudden collapse in the size of the GC after 25 and 35 nodes have been removed, which corresponds to the isolation of Alaska, as described above, the rate at which the GC collapses is much closer to the other regimes.

More interesting is the variation in the similarity of the removed node sets. Figure 5a, shows the Jaccard similarity for all combinations of the unweighted removal regimes. The similarity of the degree and VE node sets show considerable variation with multiple peaks and troughs across the full range of removed nodes. The largest variations in JC occurs up to about 75 removed nodes, these variations suggest that while the similarity of the sets is ultimately quite high with a JC of approximately 0.7, the ordering of those nodes is markedly different between the two regimes. Interestingly, the trough in JC at about 25 nodes corresponds to the plateau in network capacity observed in Fig. 3 when $2.5\%$ of the nodes have been removed. The comparison of both the degree and VE node sets with BC show a much more consistent profile. Additionally, the nodes identified by BC are more similar to those identified by VE having a JC of approximately 0.475 for a removed node set of cardinality 200, whereas the comparable node set identified by degree has a lower JC of approximately 0.35.

The similarity of the removed node sets for the weighted removal regimes are compared in Fig. 5b. The behavior of JC for weighted degree and weighted VE shows a clear variation in the node sets for about the first 50 nodes to be removed but apart from the small peak at about 25 nodes the JC exhibits a consistent upward trend to a limit of about 0.8 for node set cardinalities up to 200. The variation in JC for weighted degree and weighted VE with weighted BC are broadly similar. The smallest node sets exhibit very little overlap, with JC values increasing from 0 to about 0.3 for node sets of cardinality up to 10. As the size of the node sets increase towards 50, JC increases to a peak of approximately 0.6 for weighted VE against weighted BC and approximately 0.5 for weighted degree against weighted BC. Beyond this point the similarities for both cases plateau at approximately 0.47 and 0.42 for the weighted VE and weighted degree cases respectively.

The main conclusions to draw from this analysis is that in both its weighted and unweighted forms, VE identifies node sets that are a hybrid of those identified by degree and those identified by BC. Additionally, for cardinalities of about 50 and above, the plateaus in all the JC values suggests that all the removal regimes identify a common subset of nodes and the relative size of that subset remains roughly constant.

**Fig. 6:**
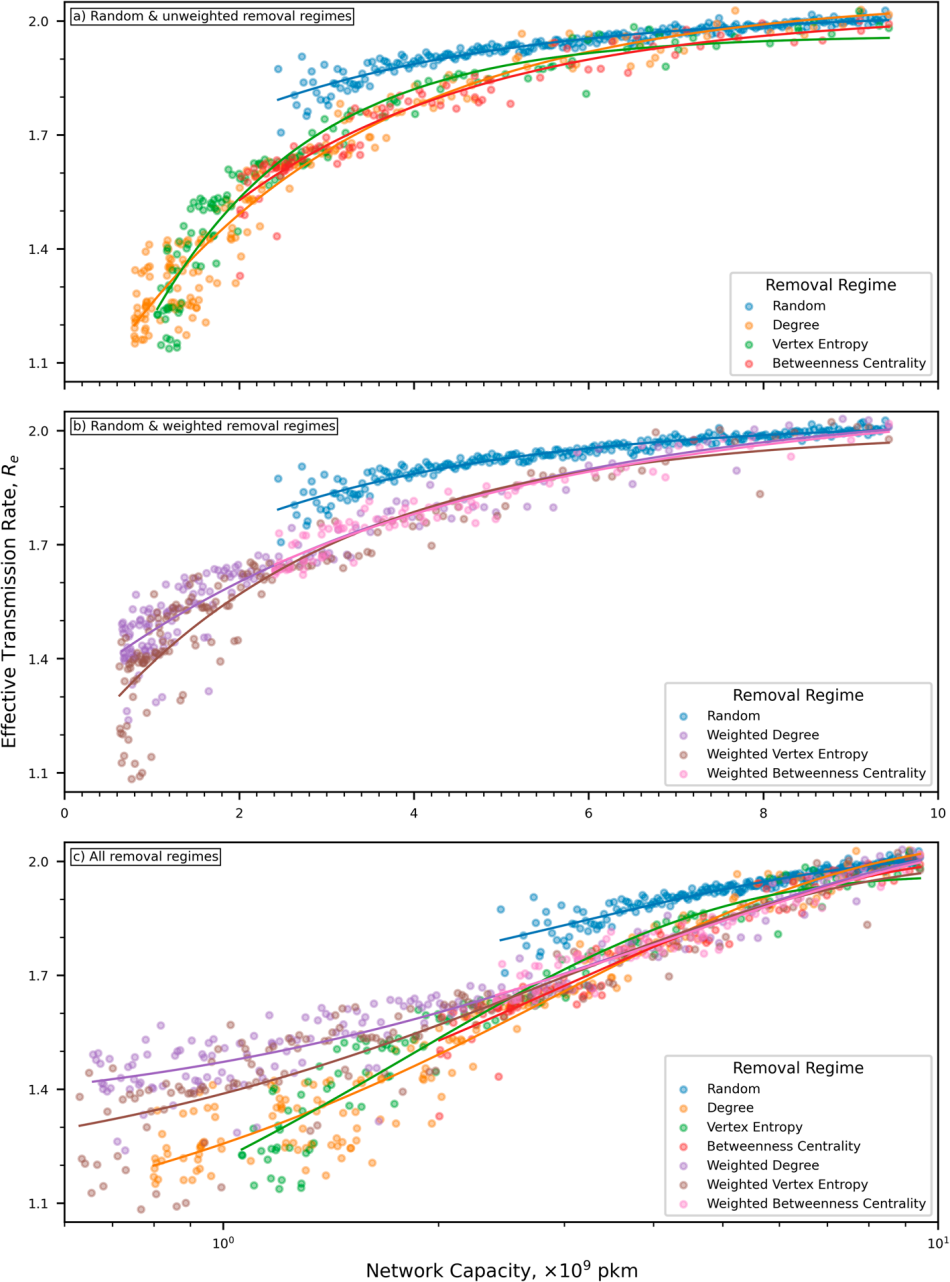
The reduction in effective transmission rate as the capacity of the airline network reduces under random and targeted node removal. **a** Compares random removal with all unweighted regimes, **b** compares random removal with the weighted regimes, and **c** compares all regimes with capacity on log scale. Targeted node removal regimes retain a higher network capacity for a given transmission rate when compared with random node removal. Above a network capacity of ${3.0\!\times \!10^9\text { pkm}}$ the behavior of all targeted regimes behave similarly. Below ${3.0\!\times \!10^9\text { pkm}}$ the behaviors diverge with the unweighted regimes exhibiting a lower transmission rate for a given network capacity

Until now our analyses have looked primarily at the network dismantling characteristics of the different node removal regimes. We now shift our focus to how epidemic transmission is impacted by those different regimes. Figure 6 shows how the effective transmission rate on the network varies with its passenger carrying capability. For clarity, we have split Fig. 6 into three subfigures. Figure 6a shows the results from the random and unweighted removal regimes, Fig. 6b compares the random and weighted removal regimes, and finally Fig. 6c show all simulations but with the network capacity displayed using a log scale. In all removal scenarios, a reduction in the capacity of the network reduces the transmission rate. Unsurprisingly, random node removal has the least impact. Indeed, even when network capacity is reduced by two-thirds, the reduction in $R_e$ is only $7.5\%$ at 1.85. At this same network capacity, all of the targeted node removal regimes show a far more substantial drop in transmission rate to about 1.7, an overall reduction of about $15\%$, double the reduction achieved by random removal. Interestingly the behaviour of $R_e$ for both BC-based removal regimes is within the performance bounds of all the other targeted removal regimes, despite the superior network dismantling capabilities discussed earlier in this section. The large reduction in the size of the GC when the Alaskan region of the airline route network gets disconnected has no material impact upon the characteristics of the epidemic spread.

An alternative analysis is to examine the capacity of the network at a target transmission rate. On inspection of Fig. 6 and for random node removal, a reduction of 0.15 in $R_e$ corresponds to a reduced network capacity of ${3.2\!\times \!10^9\text { pkm}}$. For targeted node removal, that same reduction of 0.15 in $R_e$ corresponds to a capacity of about ${5.0\!\times \!10^9\text { pkm}}$, a significant improvement given our objective of maximizing capacity whilst minimizing rate of transmission. We conclude that we can achieve the same $R_e$ but retain about $50\%$ more capacity using targeted node removal. Cross-referencing these capacities with Fig. 3 and Table 2 shows that about 650 nodes need to be removed or disconnected using random removal. Under targeted removal, and depending upon the specific regime, only about ${70\text {--}140}$ nodes need to be removed. However, if we extend our analysis and hypothesise that we need to achieve a value of 1.7 for $R_e$ the picture changes dramatically. Examination of Fig. 6 shows that for all targeted regimes a network capacity of approximately ${3.2\!\times \!10^9\text { pkm}}$ corresponds to the desired ${R_e=1.7}$. Further cross-referencing with Fig. 3 and Table 2 shows that in order to reduce network capacity to the required level, about ${180\text {--}220}$ nodes need to be removed or disconnected for the weighted and unweighted forms of degree and VE. However, for the two BC-based removal regimes approximately ${440\text {--}475}$ nodes need to be removed or disconnected, a remarkable difference. The reason for this large disparity is the sudden collapse in the GC brought about by the disconnection of the Alaskan airports with no corresponding drop in network capacity, a reason that is only valid for this experimental scenario. A more general conclusion is that despite the apparent superiority of BC as a network dismantling metric with regard to network size, a much more important consideration is the reason you wish to dismantle the network in the first place. It therefore seems reasonable to assume that the network feature you need to optimize must feature in the dismantling process.

Further examination of Fig. 6 shows that for capacities below about $3.0\!\times \!10^9\text { pkm}$ the behaviours of the targeted removal regimes diverge a little, with the unweighted forms giving larger reductions in transmission rate. This behavior is consistent with our earlier observation regarding weighted regimes retaining more flights and hence providing more opportunity for transmission to occur. We also observe that at the lowest capacities, both VE-based regimes produce larger reductions in transmission than their degree-based counterparts.

An interesting feature in the relationship of $R_e$ with capacity is revealed by Fig. 6c. For all regimes apart from VE there appears to be an inflection point at a capacity of around $3.0\!\times \!10^9\text { pkm}$. For capacities greater than this, all regimes show the same behaviour; $R_e$ increases with capacity but at a decreasing rate. Below $3.0\!\times \!10^9\text { pkm}$, a slightly different pattern emerges. For all targeting regimes, with the exception of VE, $R_e$ reduces with capacity, but at a decreasing rate. For VE however, the rate at which $R_e$ reduces appears to remain constant. This behavior could be explained by VE specifically targeting nodes that do not have a locally clustered neighborhood. Removing such nodes will preferentially isolate portions of the network with less sensitivity to the degree of the node, and therefore the likelihood of the node being a hub. This would tend to select for a more consistent reduction in capacity than targeting high capacity hubs.

**Fig. 7:**
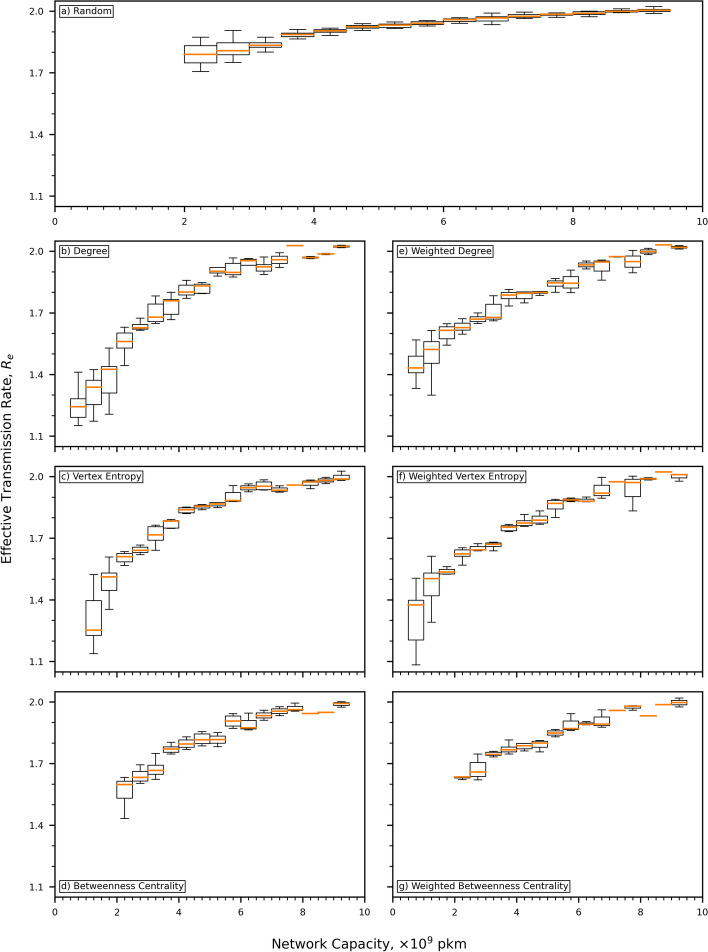
Boxplots of effective transmission rate stratified by capacity for random node removal and each of the targeted node removal regimes. **a** Shows the random removal scenario, **b**–**d** show the unweighted versions of degree, VE and BC respectively, and **e**–**g** show the weighted versions of those regimes. At capacities below ${3.0\!\times \!10^9\text { pkm}}$ there is considerable variability in the data, where it is available. The degree-based regimes exhibit the highest degree of variability. Above ${3.0\!\times \!10^9\text { pkm}}$ the VE and BC-based regimes generally display similar levels of variability

The final part of our analysis examines the distribution of effective transmission rate against stratified bins of network capacity for each node removal regime, and is shown in Fig. 7. Network capacity is stratified using bins of size $0.5\!\times \!10^9\text { pkm}$. We use a standard box and whisker plot format; the whisker lines show the range of values, the box shows the inter-quartile range, and the line shows the median value. For capacities below ${3.0\!\times \!10^9\text { pkm}}$ there is considerable variability in the values of $R_e$ with the unweighted and weighted VE regimes showing a wider distribution than the degree-based regimes. It must be noted that only the degree-based and VE-based regimes support all capacities of this magnitude, both BC-based regimes have a minimum capacity of ${2.0\!\times \!10^9\text { pkm}}$. Above capacities of ${3.0\!\times \!10^9\text { pkm}}$, the degree-based regimes exhibit the highest degree of variability. Apart from a small number of notable exceptions, for example at a capacity of ${7.75\!\times \!10^9\text { pkm}}$, the VE and BC-based regimes generally display similar levels of variability. As has been remarked before, the airline route network has been expressly built to include hubs. In the early stages of network dismantling, which corresponds to networks of relatively high capacity, the small-world effect is stronger and so the rate of transmission is dominated by the macro structure of the route network. Even if a walk begins in a remote part of the world, the small-world effect means that infected individuals will relatively quickly find their way to a hub within the network. The result of each simulation is therefore largely unaffected by the the starting point of the random walk. As dismantling continues, leaving networks of lower capacity, the impact of the small-world effect diminishes and the local structure of the route network dominates and dictates the spread of the epidemic. For example, if our walk begins in a remote location the relative absence of hubs will cause it to take longer for the epidemic to escape its starting neighborhood and spread to the rest of the network. The consequence being that the random choice of initial location for each walk becomes more important to the spread of the epidemic and introduces the higher levels of variability we observe in Fig. 7. Even with the increase in variability at low capacity, the performance of BC and VE, in both weighted and unweighted varieties is more clearly visible from these plots.

## Conclusions

In our analysis we have demonstrated that it is possible to choose a scheme for route restriction in transport networks that optimizes capacity whilst restricting the rate of epidemic spread. In particular, by using either a weighted vertex entropy or degree scoring of airports we are able to reduce the effective reproduction rate of an epidemic spread whilst preserving capacity much more effectively than with a random airport and route closure approach. Owing to simplifications in the model, we should not place too much reliance upon the absolute values of $R_e$. In relative terms, and for a reduction in $R_e$ of 0.15, targeted node removal yielded networks with $50\%$ more capacity than those restricted by random selection.

For scale free networks it is well understood that the ‘friendship paradox’ (Feld 1991) exploits high degree hubs to propagate spreading phenomena. As noted in “Theoretical considerations”, the key fact is that the airline network contains such hubs, and as the degree distribution is not homogeneous the ‘friendship paradox’ still holds. Further, although vertex entropy is defined as a function of degree, the entropy based route restriction has a very different effect upon the capacity and epidemic in the low restriction regime. Our result may expose a subtle interplay between network function and structure.

Our experimentation also encompassed the unweighted and weighted forms of betweenness centrality, a measure that in its unweighted form has well understood efficacy in network dismantling. The results here are somewhat complicated by the modular nature of the airline network, where large and low capacity subgraphs can be disconnected by the removal of a single high betweenness centrality node. While this behaviour has a dramatic impact from the perspective of network dismantling, its impact on epidemic spread is negligible. Consequently the results need to be interpreted with care. A highly efficacious network dismantling regime, does not necessarily lead to corresponding reductions in the rate of epidemic spread rate. Coupled with the fact that vertex entropy can easily, and more naturally, incorporate a weighted network and is equally effective, we believe it is more generally applicable as a metric to optimize capacity and suppress infection.

The purpose of our work is to motivate a discussion on how we can moderate our approach to extreme shutdown measures as a way to manage the public health challenges of COVID-19. We believe there are many ways to improve the robustness of our model, and in further work we intend to refine our results by taking a route by route approach to restrictions and also implementing a finer grained spreading model. In addition, there are other approaches to analyzing the effect of graph structure on epidemic spread, such as spectral radius of the adjacency matrix (Draief et al. 2006). It is possible that these results could provide further analytical tools to link the dismantling metrics directly to epidemic thresholds, which we anticipate could be interesting lines of further inquiry. In particular, we have not constrained the route restriction by other criteria such as retaining, or conversely severing, communications between particular geographic regions. These additional constraints would likely change radically the conclusions of our experiments. Despite the acknowledged shortcomings, we feel that our result justifies further exploration.

## Data Availability

The source data analysed during this study are available at the OAG Yearly Historic Flight Schedules (10.7910/DVN/COHFWA), and from ourairports.com (https://ourairports.com/data/airports.csv). The data supporting the conclusions of this study are available from the corresponding author upon reasonable request.

## Abbreviations

BC: Betweenness centrality
GC: Giant component
JC: Jaccard similarity coefficient
pkm: Passenger kilometers
SIR: Susceptible-infected-recovered
VE: Vertex entropy
